# MicroRNA-4476 promotes glioma progression through a miR-4476/APC/β-catenin/c-Jun positive feedback loop

**DOI:** 10.1038/s41419-020-2474-4

**Published:** 2020-04-23

**Authors:** Jie Lin, Shengfeng Ding, Cheng Xie, Renhui Yi, Zhiyong Wu, Jie Luo, Tengyue Huang, Yu Zeng, Xizhao Wang, Anqi Xu, Jianqi Xiao, Ye Song, Xian Zhang

**Affiliations:** 1 Department of Neurosurgery, Nanfang Hospital, Southern Medical University, 510515 Guangzhou, Guangdong PR China; 2 Department of Neurosurgery, The First Affiliated Hospital of Gannan Medical University, 341000 Ganzhou, Jiangxi PR China; 3 Department of Neurosurgery, Shanghai Tenth People’s Hospital, Tongji University School of Medicine, 200072 Shanghai, PR China; 4 Department of Neurosurgery, The First Hospital of Quanzhou Affiliated to Fujian Medical University, 362000 Quanzhou, Fujian Province PR China; 5 Department of Neurosurgery, The First Hospital of Qiqihar City, 161005 Qiqihar, PR China

**Keywords:** Tumour biomarkers, miRNAs, Prognostic markers

## Abstract

Glioma has been a major healthcare burden; however, the specific molecular regulatory mechanism underlying its initiation and progression remains to be elucidated. Although it is known that many miRNAs are involved in the regulation of malignant phenotypes of glioma, the role of miR-4476 has not been reported yet. In the present study, we identify miR-4476 as an upregulated microRNA, which promotes cell proliferation, migration, and invasion in glioma. Further mechanistic analyses indicate that the adenomatous polyposis coli (APC), a negative regulator of the Wnt/β-catenin signaling pathway, is a direct target of miR-4476 and mediates the oncogenic effects of miR-4476 in glioma. C-Jun, a downstream effector of the Wnt/β-catenin signaling, is upregulated by miR-4476 overexpression. In turn, c-Jun could positively regulate miR-4476 expression by binding to the upstream of its transcription start site (TSS). Furthermore, in our clinical samples, increased miR-4476 is an unfavorable prognostic factor, and its expression positively correlates with c-Jun expression but negatively correlates with that of APC. In conclusion, our study demonstrates that miR-4476 acts as a tumor enhancer, directly targeting APC to stimulate its own expression and promoting the malignant phenotypes of glioma.

## Introduction

Gliomas are the most frequent primary tumors of the central nervous system (CNS). They represent 81% of all malignant brain tumors, and are associated with significant morbidity and mortality. According to the 2016 version of the World Health Organization (WHO) classification of CNS tumors, gliomas can be separated into circumscribed gliomas (WHO Grade I) and diffusely infiltrating gliomas (WHO Grades II–IV) based on their growth pattern and isocitrate dehydrogenase (IDH) mutation status. Glioblastoma (GBM, WHO Grade IV) is the most malignant primary brain tumor, and accounts for approximately 45% of all gliomas^1,2^. The current standard care for GBM patients, including surgical resection followed by adjuvant radiation therapy and chemotherapy with temozolomide, produces a poor 5-year survival rate of 9.8% and median survival of only 15 months^3–5^. Owing to the heterogeneity of glioma cells, it is almost impossible to completely eliminate residual tumors with adjuvant therapy^6^. Additionally, glioma cells tend to diffusely infiltrate adjacent brain parenchyma and develop resistance to standard treatment^7,8^. Therefore, a better understanding of glioma initiation and progression is required to identify novel prognostic molecular markers as well as to develop novel therapeutic strategies for the treatment of glioma.

The Wnt/β-catenin signaling pathway regulates numerous biological behaviors throughout both development and the adult life of all animals. However, it is also frequently dysregulated in malignant epithelial cells and represents a valid target in cancer drug development^9,10^. Adenomatous polyposis coli (APC) is an essential negative regulator of Wnt/β-catenin signaling, and mutations in APC lead to the nuclear accumulation of β-catenin, which triggers the transcription of downstream genes and promotes colorectal tumorigenesis^11,12^. Mutations in Wnt/β-catenin pathway members are commonly found in many cancers, including gastrointestinal cancers, thyroid carcinoma, medulloblastoma, and gliomas^13–16^. Aberrant activation of Wnt/β-catenin signaling promotes glioma progression^17,18^. In contrast, inhibition of the Wnt/β-catenin pathway seems to elicit the opposite effect. For example, inhibition of Wnt signaling prevents temozolomide resistance by regulating O-6-methylguanine-DNA methyltransferase (MGMT) gene expression in gliomas^19^. All these studies indicate the therapeutic potential of targeting Wnt/β-catenin signaling for the treatment of gliomas.

MicroRNAs (miRNAs) are short, non-coding RNAs of approximately 22 nucleotides that regulate gene expression through sequence-specific base-pairing with their target mRNAs^20^. Recent studies have shown that aberrant expression of numerous miRNAs play important roles in the initiation and progression of several tumors^21–23^, including gliomas^24–26^, indicating that therapies targeting miRNAs may be suitable alternative strategies for the treatment of gliomas. Several miRNAs have been reported to activate Wnt/β-catenin signaling and promote cancer progression by targeting APC^27,28^. MiRNA-4476 expression has been found abnormal in pancreatic and biliary-tract cancers^29^. Nevertheless, whether dysregulated miRNA-4476 expression has a role in gliomagenesis or glioma progression, and whether this role is mediated by APC interaction in glioma, have not been reported.

In this study, we found that upregulation of miR-4476 in human gliomas is correlated with tumor progression, and overexpression of miR-4476 promotes the proliferation, migration, and invasion of glioma cells through a miR-4476/APC/β-catenin/c-Jun positive feedback loop.

## Materials and methods

### Cell culture and collection of clinical glioma tissue samples

The human glioma cell lines U87 and LN229 were purchased from the Chinese Academy of Sciences (Shanghai, China) and cultured in Dulbecco’s modified Eagle’s medium (DMEM) (Biological Industries, Israel) supplemented with 10% fetal calf serum (Hyclone, Logan, UT, USA) at 37 °C in a humidified atmosphere of 5% CO_2_. Sources of both cell lines were identified and verified by STR profiling. No mycoplasma contamination was found in both cell lines.

All glioma samples and nontumor brain samples were obtained from the Department of Neurosurgery, Nanfang Hospital of Southern Medical University, Guangzhou, China. Nontumor brain tissue samples were derived from patients who underwent brain trauma surgery. Nine nontumor brain samples and thirty-six glioma samples were used to extract total RNA. A total of 87 paraffin-embedded glioma samples were used for immunohistochemical staining and in situ hybridization. All patients have provided prior consent for the use of these clinical tissue samples for research purposes, and ethical approval was obtained from the Ethics Committees of Nanfang Hospital. All specimens had confirmed pathological diagnosis and were classified according to WHO criteria.

### Real-time quantitative PCR

Total RNA was extracted from glioma cell lines, glioma tissues, and normal brain tissues using Trizol (Takara Bio, Shiga, Japan). The U6 and ARF5 genes were used as miRNA and gene internal controls, respectively. Cycling conditions were 95 °C for 10 min to activate the DNA polymerase, followed by 45 cycles of 95 °C for 15 s, 60 °C (for miR-4476, APC, and c-Jun) for 15 s, and 72 °C for 10 s. Amplicon specificity was confirmed by melting curve analysis. Each independent experiment was performed in triplicate. MiR-4476- and gene-specific (APC, c-Jun, and ARF5) primer sequences are shown in Supplementary Table 1.

### Transient transfection

MiR-4476 mimics and inhibitors, negative control, and three siRNAs targeting APC, were designed and synthesized by Guangzhou RiboBio (RiboBio Inc., China). The most effective siRNA identified by qPCR was used for subsequent experiments. Twelve hours before transfection, GBM U87 or LN229 cells were plated onto 6- or 96-well plates (SORFA, China) at 30–50% confluence. Lipofectamine 2000 Transfection Reagent (Invitrogen, Beijing, China) was then used to transfect siRNA, mimics, or inhibitors into cells according to the manufacturer’s protocol. Cells were collected after 48–72 h for further experiments. Specific siRNA, mimics, or sense inhibitors for miR-4476 and APC are shown in Supplementary Table 2.

### Lentivirus production and infection

Lentiviral particles carrying the hsa-miR-4476 precursor vector and the control lentiviral vector were constructed by GeneChem (Shanghai, China). U87 cells were infected with the lentiviral vector, and GFP-positive polyclonal cells were selected for further experiments.

### Western blot analysis

Western blot was performed as previously described^30^. Antibodies included anti-APC, anti-c-Jun, anti-c-Myc, anti-CCND1, anti-β-catenin, anti-Snail, anti-Slug, anti-E-cadherin, anti-N-cadherin, anti-β-actin, anti-β-tubulin, and anti-GAPDH. The antibodies are listed in Supplementary Table 3. The images were captured with a Minichemi 910 Plus RGB (Sage, Beijing, China). Signals were detected using enhanced chemiluminescence reagents (Millipore, USA).

### Immunohistochemical staining

Paraffin sections prepared from in vivo experiments and clinical tissue samples were used for immunohistochemistry assays to detect the protein expression levels of APC, c-Jun, and Ki-67. The indirect streptavidin-peroxidase method was used as previously described^24^. Immunohistochemically stained tissue sections were examined separately by two pathologists. The antibodies used are listed in Supplementary Table 3.

### In situ hybridization

Tissue sections were dewaxed in xylene, rehydrated through an ethanol gradient, and then treated with 3% H_2_O_2_ for 10 min. Sections were treated with diluted pepsin in 3% fresh citrate buffer at 37 °C for 30 min and then washed. Hybridization with digoxigenin (DIG)-labeled miRCURY LNA probes (Axl-bio, Guangzhou, China) was performed overnight at 37 °C after prehybridization in 20 mL of a prehybridization solution for 2 h at 37 °C. After hybridization, sections were subjected to high stringency washes with 2× SSC, 0.5× SSC, and 0.2× SSC for 5, 15, and 15 min, respectively, at 37 °C. Sections were subsequently incubated in blocking solution for 30 min at 37 °C and then with alkaline phosphatase-conjugated sheep anti-DIG Fab fragments for 60 min at room temperature. Positive miR-4476 staining was observed by adding BM purple alkaline phosphatase substrate (Roche, Basel, Switzerland) according to the manufacturer’s instructions.

### Cell viability and proliferation assay

Cell proliferation was analyzed by MTT assay. Cells were plated in 96-well plates at a density of 1000–2000 cells/well and incubated for 24 h. Approximately 20 μL of MTT (5 mg/mL) (Sigma, USA) was added into each well and incubated for 4 h. The supernatants were then removed, and 150 μL of dimethyl sulfoxide (DMSO; Sigma) was added to each well. The absorbance value (OD) of each well was measured at 490 nm at the same time for the next 4 days. Five wells were used as replicates for each experimental condition.

### EdU proliferation assay

Proliferating U87 and LN229 cells were examined using the Cell-Light™ EdU Apollo®567 In Vitro Imaging Kit (RiboBio Inc.) according to the manufacturer’s protocol. Cells were incubated with 10 μM EdU for 2 h before fixation in 4% paraformaldehyde, permeabilization in 0.3% Triton X-100, and EdU staining. Nuclei were stained with 5 μg/mL DAPI for 10 min. EdU-positive cells were counted under a microscope in five random fields (×200). All assays were performed in triplicate independently.

### In vivo tumorigenesis in nude mice

A total of 1 × 10^6^ U87 cells transfected with LV-miR-4476 or the negative control lentiviral vector (*N* = 5 per group) in 100 μL DMEM without FBS were subcutaneously injected into the dorsal flank of 6-week-old male BALB/c nude mice. Sample size was estimated based on our preliminary experimental results. All mice were maintained in a barrier facility on HEPA-filtered racks and fed an autoclaved laboratory rodent diet. Tumor size was measured every 3 days post-implantation. After 21 days, all mice were euthanized, and tumor tissues were excised and weighed. The investigators were completely blinded to the allocation when measuring the size and weight of tumor tissues. No mouse was excluded in the analysis. All animal studies were conducted in accordance with the principles and procedures outlined in the National Institutes of Health Guide for the Care and Use of Animals under assurance number A3873-1.

### Transwell and Boyden assays

Transwell and Boyden assays were performed according to our previous study^24^. For the cell migration assay, 5 × 10^4^ cells in 100 μL of DMEM medium without FBS were plated into the upper chamber of a Transwell apparatus (Corning, USA). DMEM (500 μL) supplemented with 10% FBS was added as a chemoattractant in the lower chamber. After the cells had been incubated for 6 h at 37 °C in a 5% CO_2_ atmosphere, the insert was washed with PBS, and cells on the top surface of the insert were wiped with a cotton swab. Cells adhering to the lower surface were fixed in methanol, stained with crystal violet solution, and counted under a microscope in 3 predetermined fields (×200). All assays were independently repeated at least three times. The procedure for the Boyden assay was similar to that for the Transwell assay, except that the Transwell membranes were first precoated with 24 μg/μL Matrigel (R&D Systems, USA) and the cells were incubated for 10 h at 37 °C in a 5% CO_2_ atmosphere. Cells adhering to the lower surface were counted as for the cell migration assay.

### Luciferase reporter assays

To confirm that APC is a direct target of miR-4476, a 284-bp fragment of the APC 3′-UTR was cloned into the psiCHECK-2 vector (named WT). Site-directed mutagenesis of the miR-4476 binding site in the APC 3′-UTR (named MT) was performed using the GeneTailor Site-Directed Mutagenesis System (Invitrogen, Beijing, China). For reporter assays, the WT or MT vector and the control psiCHECK-2 vector were co-transfected into HK293T cells with miR-4476 mimics or inhibitors in 48-well plates. The cells were then harvested for luciferase assay 48 h after transfection. Luciferase assays were performed using the Dual-Luciferase Reporter Assay kit (Promega Corporation, Madison, WI, USA) according to the manufacturer’s protocol.

To investigate the effects of miR-4476 on the activity of the Wnt/β-catenin signaling pathway, Topflash (harboring three optimal TCF-binding sites) or Fopflash (harboring three mutated TCF-binding sites) luciferase reporter were co-transfected into HK293T cells with miR-4476 mimics or inhibitors in 48-well plates. The cells were then harvested for luciferase assay 48 h after transfection. Luciferase assays were performed using the Dual-Luciferase Reporter Assay kit (Promega Corporation, Madison, WI, USA) according to the manufacturer’s protocol.

To confirm that miR-4476 is a direct target of c-Jun, a fragment of the miR-4476 promoter was cloned into the pGL3-basic vector. For luciferase reporter assays, the vector containing the miR-4476 promoter and the control pGL3-basic vector were co-transfected into HK293T cells with a c-Jun overexpression plasmid in 48-well plates. All cells were harvested for luciferase assay 48 h after transfection. Luciferase assays were performed using the Dual-Luciferase Reporter Assay kit (Promega Corporation, Madison, WI, USA) according to the manufacturer’s protocol.

### Chromatin immunoprecipitation assay

DNA-protein complexes were immunoprecipitated from U87 and LN229 cells using the Chromatin Immunoprecipitation Kit (Millipore) according to the manufacturer’s protocol, with anti-c-Jun and normal mouse IgG polyclonal antibodies (CST, USA), the latter serving as a control for nonspecific DNA binding. The precipitated DNA was subjected to qPCR analysis using gene-specific primers. The primers were utilized to amplify across the miR-4476 promoter region (Supplementary Table 1).

### Electrophoretic mobility shift assay

Binding activity on the miR-4476 promoter region was determined using the EMSA Kit (Biosense Bioscience Co., Ltd, Guangzhou, China) according to the manufacturer’s protocol. The preformed c-Jun recognized probe (Axl-bio) was used as positive control. Samples without nucleoprotein were used as negative controls. For competition experiments, a 100-fold excess of specific competitor oligonucleotide (unlabeled wild-type c-Jun probes) was added to the binding mixture 10 min before adding the labeled probe. Visualized bands were analyzed using Tanon-4100 (Tanon, Shanghai, China). The sequences used are listed in Supplementary Table 1.

### Statistical analysis

All quantified data represent an average of at least triplicate samples. SPSS 24.0 (SPSS Inc. Chicago, IL, USA) and GraphPad Prism 6.0 (GraphPad software, La Jolla, CA, USA) were used for statistical analysis. Data are presented as means ± SD. One-way ANOVA (analysis of variance) or two-tailed Student’s *t*-test was used for comparisons between groups. MTT results were analyzed by two-way ANOVA. Chi-square or Fischer’s exact test was used to identify differences between categorical variables. Associations between miR-4476 and APC, miR-4476 and c-Jun, or APC and c-JUN, were analyzed using a Chi-square test. Survival analysis was performed using the Kaplan–Meier method. Differences were considered significant when *p* < 0.05. All statistical tests were two-sided, and single, double, triple and quadruple asterisks indicate statistical significance, *p < 0.05, **p < 0.01, ***p < 0.001, and ****p < 0.0001.

## Results

### MiR-4476 is upregulated in glioma and promotes glioma cell proliferation, migration, and invasion in vitro

**Fig. 1:**
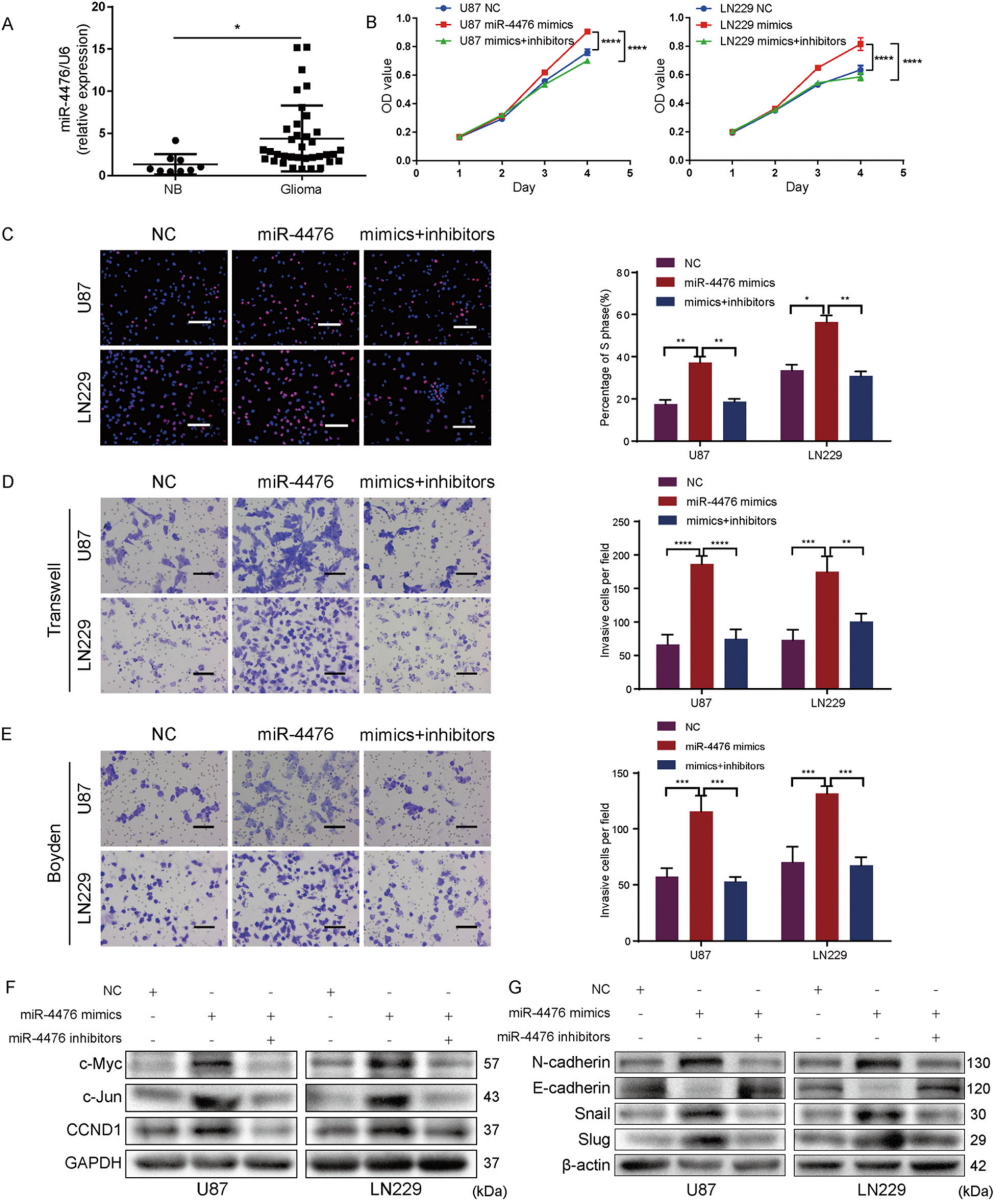
MiR-4476 is overexpressed in glioma and promotes glioma cell proliferation, migration, and invasion in vitro. **a** qPCR analysis of miR-4476 expression in nontumor brain tissues (NB) and glioma tissues. **b** The cell viability of U87 and LN229 cells transfected with miR-4476 mimics or inhibitor was evaluated by the MTT assays. **c** The proliferation ability of U87 and LN229 cells was evaluated by EdU assays; scale bar = 50 μm. **d**, **e** The migration ability and invasion ability of U87 and LN229 cells were tested by Transwell (**d**) and Boyden (**e**) assays, respectively; scale bar = 75 μm. **f**, **g** Western blot analysis examined the expression of crucial cell-cycle (**f**) and EMT (**g**) related proteins. Mean ± S.D., *p < 0.05, **p < 0.01, ***p < 0.001, and ****p < 0.0001.

To identify the role of miR-4476 in glioma progression, we first compared its expression level between nontumor brain tissues (NB) and glioma tissues by quantitative PCR (qPCR) (Fig. 1a). We found that miR-4476 expression was upregulated in glioma when compared with nontumor brain tissues.

To further explore the biological function of miR-4476 in glioma cells, miR-4476 mimics or inhibitors were transfected into U87 and LN229 cells. Results of MTT (Fig. 1b) and EdU incorporation assays (Fig. 1c) showed that overexpression of miR-4476 markedly promoted cell growth and G1 to S phase cell-cycle transition in both cell lines. Transwell (Fig. 1d) and Boyden assays (Fig. 1e) further showed that miR-4476 mimics significantly enhanced the migratory and invasive ability of glioma cells. However, all these biological effects could be mitigated by transfection of miR-4476 inhibitors.

To elucidate the underlying mechanisms by which miR-4476 promotes glioma cell proliferation, migration, and invasion, cell-cycle- and epithelial–mesenchymal transition (EMT)-related proteins were examined by western blot (Fig. 1f, g). We found that miR-4476 overexpression enhanced c-Jun, c-Myc, G1/S-specific cyclin-D1 (CCND1), Snail, Slug, and N-cadherin expression levels, but suppressed that of E-cadherin, while miR-4476 inhibitors reversed all these effects of the mimics.

### MiR-4476 significantly enhances xenografted tumor growth in vivo

**Fig. 2:**
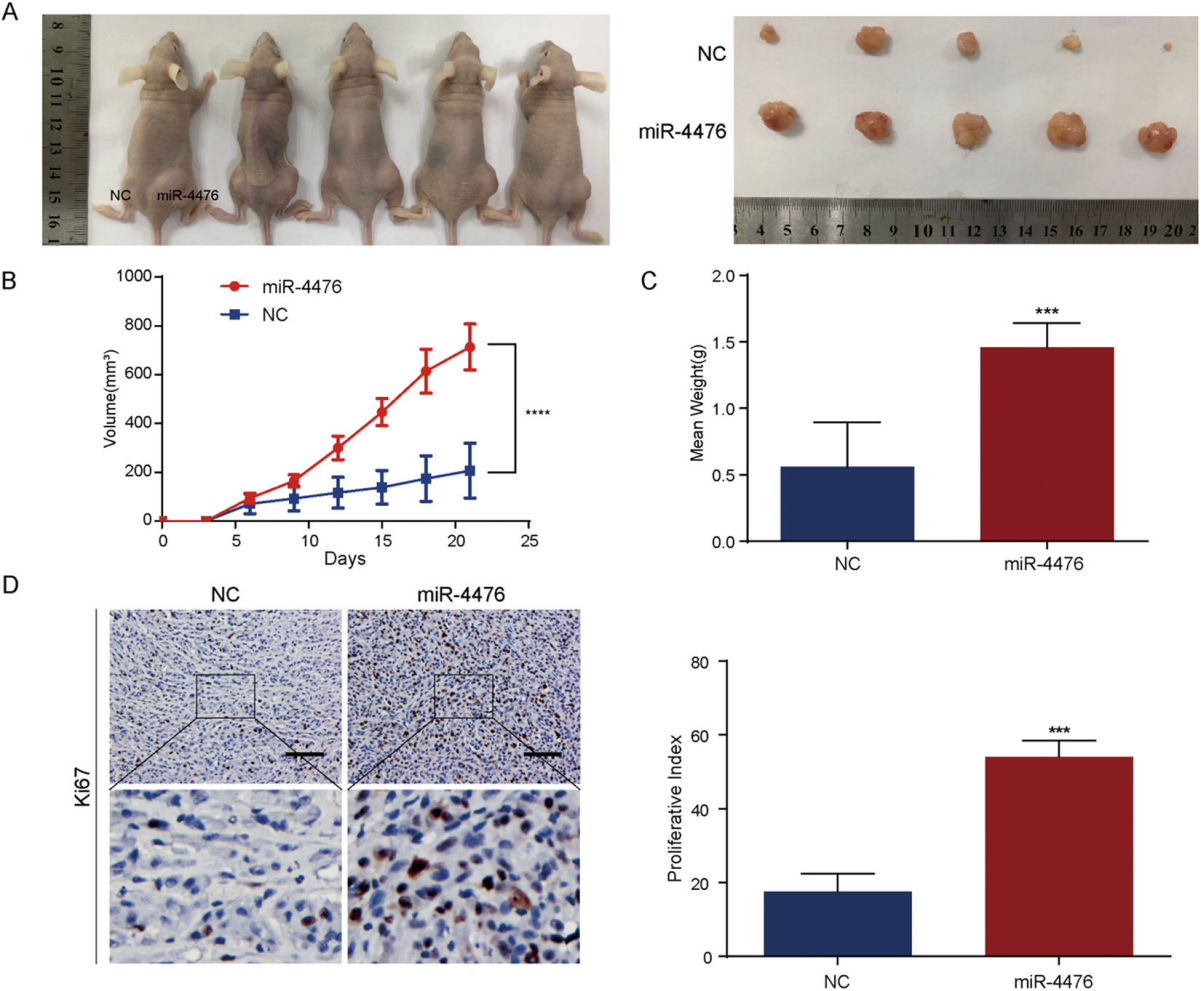
MiR-4476 promotes xenograft tumor growth in vivo. **a** Images of xenograft tumor models of mice injected with U87 cells transfected with miR-4476 lentiviral expression particles or negative control (NC) lentiviral expression particles. **b** Tumor volume was periodically measured for each mouse and tumor growth curves were plotted. **c** Tumor weight was measured at the 21st day after inoculation in each group. **d** IHC staining was used to detect and quantify Ki-67 expression in xenograft tumors from mice injected with U87 cells; scale bar = 75 μm. Mean ± S.D., *p < 0.05, **p < 0.01, ***p < 0.001, and ****p < 0.0001.

U87 cells were infected with lentiviral particles carrying the hsa-miR-4476 precursor vector or control lentiviral vector, and overexpression of miR-4476 was confirmed by qPCR (Supplementary Fig. 1A). GFP-positive polyclonal cells were selected for further experiments using fluorescence-activated cell sorting (Supplementary Fig. 1B). Next, in vivo tumor formation was induced by subcutaneous implantation of U87-miR-4476 and U87-NC (negative control) cells into nude mice. Tumor size was measured every 3 days, and all the mice were sacrificed 21 days after implantation. As shown in Fig. 2a–c, xenografted tumors derived from U87-miR-4476 cells exhibited a higher growth rate and greater tumor burden than those in the control group. Moreover, staining for Ki-67 showed a greater number of proliferative cells in the miR-4476 overexpression group than in the control group. These results suggested that miR-4476 upregulation markedly enhances glioma cell proliferation in vivo.

### MiR-4476 directly targets APC and activates the Wnt/β-catenin signaling pathway

**Fig. 3:**
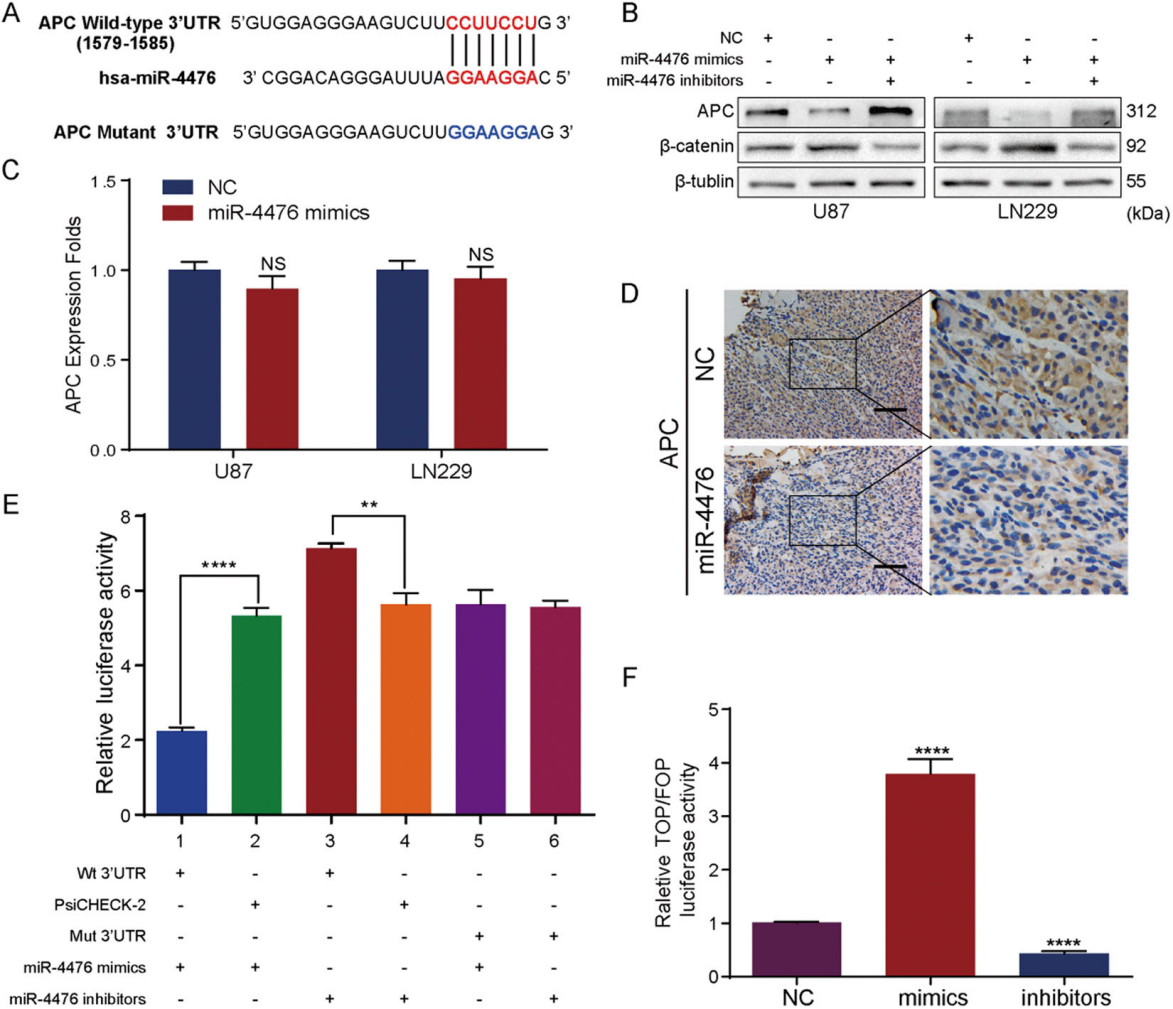
MiR-4476 directly targets APC and activates Wnt/β-catenin signaling pathway. **a** miR-4476 and its putative binding sequences in the 3’UTR of APC. A mutation was generated in the complementary site that binds to the seed region of miR-4476. **b** Western blot demonstrated downregulated APC level under miR-4476 mimics transfection, whereas co-transfection with miR-4476 inhibitor reversed this effect. **c** APC mRNA expression was detected by QPCR in miR-4476-overexpressing U87 and LN229 cells. **d** APC expression was evaluated by IHC in xenografts derived from U87 cells; scale bar = 75 μm. **e** Luciferase reporter assay was used to determine miR-4476 direct targeting the APC 3’UTR. **f** TOPFlash/FOPFlash reporter assay was used to investigate the influence of miR-4476 in Wnt/β-catenin signaling. Mean ± S.D., *p < 0.05, **p < 0.01, ***p < 0.001, and ****p < 0.0001. wt, wild-type; mut, mutant.

Using bioinformatic analysis, we identified a putative binding site for miR-4476 within the 3′-UTR of human APC mRNA (Fig. 3a). Western blot analysis showed that miR-4476 overexpression led to reduced APC expression, whereas miR-4476 inhibitors could rescue this suppression (Fig. 3b). Interestingly, qPCR analysis showed that miR-4476 overexpression had little effect on APC mRNA levels (Fig. 3c), indicating that miR-4476 may inhibit APC through a post-transcriptional mechanism. Consistent with the in vitro results, immunohistochemical staining of xenograft tissues revealed a marked reduction in APC expression in the miR-4476 overexpression group, as compared to the control group (Fig. 3d). To confirm that APC is a direct target of miR-4476, the 3′-UTR of APC containing the putative miR-4476 binding site was cloned into a luciferase expression vector. Co-transfection of miR-4476 mimics and a reporter vector containing the wild-type APC 3′-UTR into HK293T cells significantly decreased luciferase reporter activity, whereas co-transfection of miR-4476 inhibitors had the opposite effect (Fig. 3e). These effects on luciferase activity were abrogated when HK293T cells were co-transfected with a reporter vector containing a mutated APC 3′-UTR. These data confirmed that APC is indeed a direct target of miR-4476.

Because APC is an essential component and negative regulator of the Wnt/β-catenin signaling pathway and miR-4476 is a negative regulator of APC, we reasoned that miR-4476 could activate Wnt/β-catenin signaling by directly targeting APC. Western blot analysis confirmed that transfection of miR-4476 mimics resulted in increased expression of β-catenin, while co-transfection with miR-4476 inhibitors reversed the effects of the mimics (Fig. 3b). Next, we used a TOPFlash/FOPFlash reporter assay to investigate the effect of miR-4476 on Wnt/β-catenin signaling (Fig. 3f). Transfection of the miR-4476 mimics significantly increased Top/Fop transcriptional activity, whereas transfection of miR-4476 inhibitors elicited the opposite effect.

Together, these results suggest that miR-4476 can activate the Wnt/β-catenin signaling pathway by directly targeting APC.

### Silencing APC mitigates the suppression of malignant phenotypes induced by miR-4476 inhibitor

**Fig. 4:**
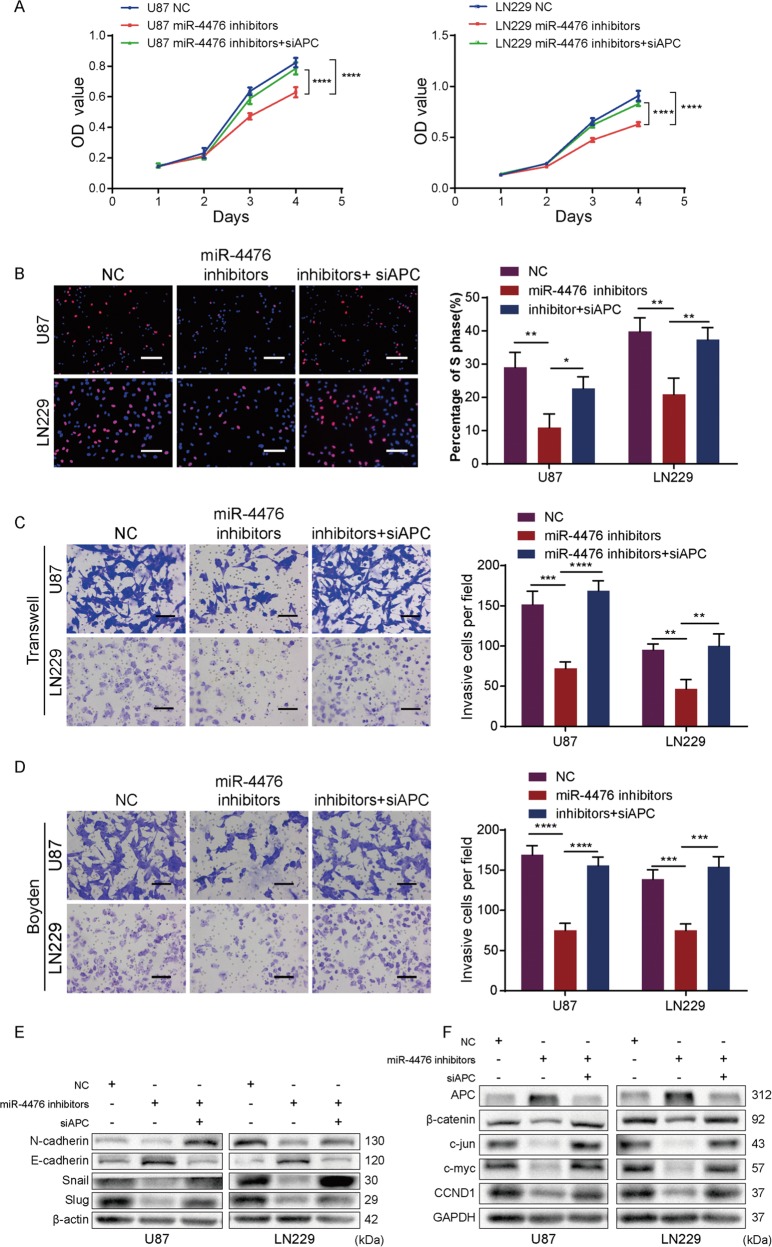
Silencing APC mitigates the suppression effects of miR-4476 inhibitor. **a**, **b** MTT assays (**a**) and EdU assays (**b**) showed miR-4476 inhibitor suppressed U87 and LN229 cells proliferation and silencing APC reversed these effects; scale bar = 50 μm. **c**, **d** Transwell assays (**c**) and Boyden assays (**d**) showed silencing APC restored the migration and invasion ability suppressed by miR-4476 inhibitor in U87 and LN229 cells; scale bar = 75 μm. **e**, **f** Western blot of endogenous N-cadherin, E-cadherin, snail, slug, APC, β-catenin, c-Jun, c-myc, and CCND1 protein expression levels in U87 and LN229 cells treated as indicated. β-actin and GAPDH served as loading controls. Mean ± S.D., *p < 0.05, **p < 0.01, ***p < 0.001, and ****p < 0.0001.

Three small-interfering RNAs (siRNAs) targeting APC were used to silence APC expression in U87 and LN229 cells (Supplementary Fig. 1C). As we found that siAPC2 was the most effective at silencing APC, this siRNA was selected for subsequent experiments. Consistent with the abovementioned results, MTT, EdU, Transwell, and Boyden assays demonstrated that miR-4476 inhibitors could suppress the proliferation, migration, and invasion of U87 and LN229 cells, and these changes could be reversed by silencing APC (Fig. 4a–d). Furthermore, western blot showed miR-4476 inhibitor suppressed both EMT (Fig. 4e) and Wnt/β-catenin/cell-cycle pathway activity (Fig. 4f) in glioma cells, while co-transfection with APC siRNA reversed these effects.

These results suggested that miR-4476 promotes glioma progression by activating the Wnt/β-catenin signaling pathway, and APC is a critical mediator in this process.

### C-Jun upregulates miR-4476 expression by binding to its promoter region

**Fig. 5:**
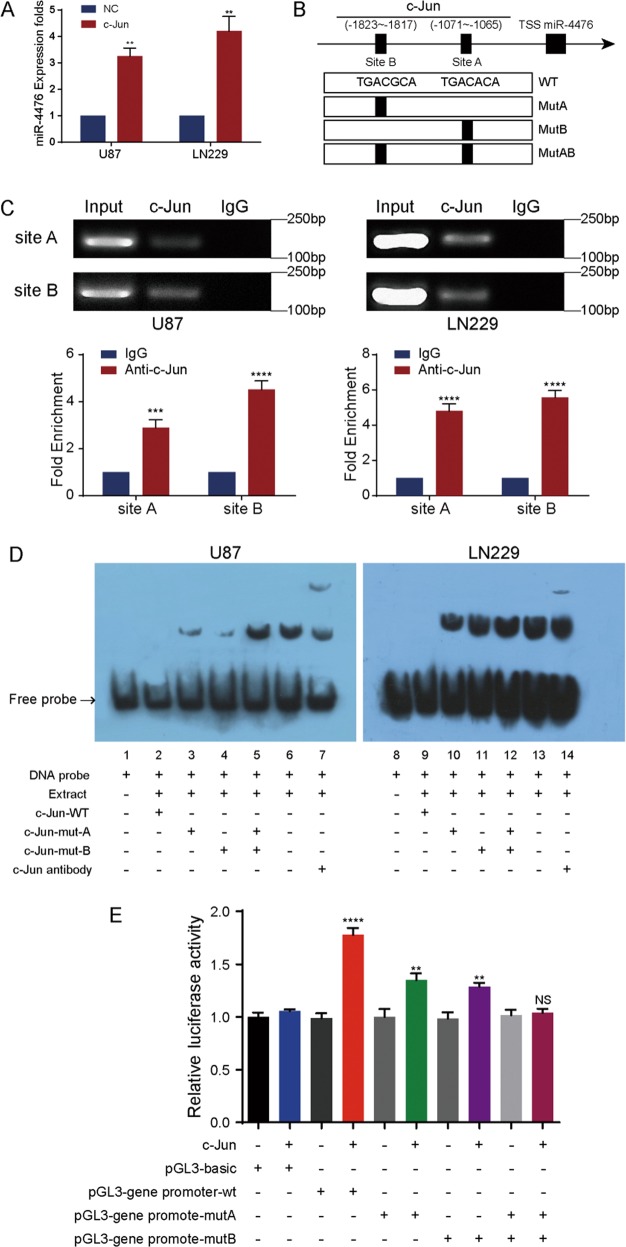
C-Jun upregulates miR-4476 by binding to its promoter region. **a** C-Jun overexpression upregulated miR-4476 confirmed by QPCR assays. **b** Schematic representation of the promoter regions of miR-4476 with the putative c-Jun TFBSs (site A and site B) and the structure of the wild-type (WT) and TFBS mutant (MutA, MutB, and MutAB) luciferase reporters driven by the promoter. **c** ChIP-QPCR and PCR gel showed there was significant c-Jun enrichment in the specific regions containing the two predicted binding sites. **d** EMSA result was shown from nuclear proteins extracted from U87 and LN229 cells after incubation with individual DIG-ddUTP-labelled oligonucleotide probes (lanes 2–6, 9–13). The free probe of labelled c-Jun was run in lanes 1 and 8 as a control. A 100-fold excess of unlabelled c-Jun-WT was used to compete with c-Jun binding (lanes 2 and 9, compared with lanes 6 and 13). A 100-fold excess of unlabeled mutated c-Jun-A and c-Jun-B was used to compete with binding of respective labelled probes (lanes 3–5 and lanes 10–12 compared with lanes 2 and 8). **e** Relative luciferase activity of the indicated promoter vectors in 293 T cells transfected with c-Jun plasmids. Mean ± S.D., *p < 0.05, **p < 0.01, ***p < 0.001, and ****p < 0.0001.

C-Jun is a classic transcript factor which has been reported to regulate many miRNAs’ expression, and we have been interested in the regulation of miRNAs by c-Jun in glioma. MiRNA microarray analysis was used to identify differentially expressed miRNAs between c-Jun silencing group and control group, and miR-4476 was found to be the most differentially expressed miRNA (supplementary Table 4). Next, we overexpressed c-Jun in U87 and LN229 cells by transient transfection of a c-Jun-expressing plasmid (Supplementary Fig. 1D). QPCR analysis confirmed that miR-4476 was significantly upregulated by c-Jun overexpression (Fig. 5a), indicating that c-Jun is an upstream regulator of miR-4476.

To investigate the mechanism underlying the transcriptional regulation of miR-4476 expression by c-Jun, we analyzed a 2-kb region upstream of the transcription start site (TSS) of miR-4476 using the PROMO and JASPAR bioinformatics websites. Two c-Jun binding motifs, located at −1823~−1817bp (site A) and −1071~−1065 bp (site B) upstream of the TSS, were identified within the putative promoter region of miR-4476 (Fig. 5b).

To determine whether endogenous c-Jun binds to the miR-4476 promoter, we then performed a chromatin immunoprecipitation (ChIP) assay in U87 and LN229 cells. Indeed, there was significant c-Jun enrichment in the regions containing the two predicted binding sites (Fig. 5c).

Electrophoresis mobility shift assay (EMSA) further confirmed that c-Jun could bind the two sites (Fig. 5d). A migrating complex appeared when U87 and LN229 cell nuclear extracts were incubated with a biotin-labeled probe (lanes 6 and 13), whereas complex formation could not be observed when unlabeled, wild-type probe was added as a binding competitor (lanes 2 and 9). The complex was not significantly affected by Mut A or Mut B or Mut AB (lanes 3–5 and lanes 10–12), but it was supershifted by an anti-c-Jun antibody (lanes 7 and 14).

Furthermore, upregulation of c-Jun increased the luciferase activity of the wild-type miR-4476 promoter in the HK293T cell line (Fig. 5e). A similar effect was observed when either site A or site B were mutated, but not when both sites were mutated. These results proved that c-Jun enhances transcriptional activity of miR-4476 promoter.

Combined, our data suggest that c-Jun promotes miR-4476 transcription, and that the two identified c-Jun binding sites are both functional.

### Clinical evidence for a miR-4476/APC/β-catenin/c-Jun positive feedback loop

**Fig. 6:**
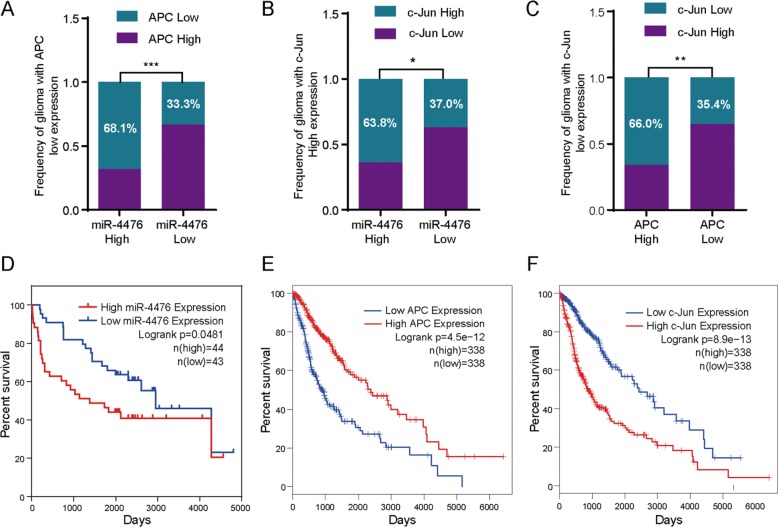
Clinical evidences of miR-4476/APC/β-catenin/c-Jun axis. **a**–**c** Expression levels correlation between 4476 and APC, miR-4476 and c-Jun, APC and c-Jun. c-Jun and APC expression level were scored by IHC staining, and miR-4476 expression levels were detected by in situ hybridization. **d** Kaplan–Meier survival analysis of overall survival of 87 glioma patients on the basis of miR-4476 expression levels. **e**, **f** Kaplan–Meier survival analysis of overall survival of 676 glioma patients on the basis of APC or c-Jun expression levels, using GEPIA website. Mean ± S.D., *p < 0.05, **p < 0.01, ***p < 0.001, and ****p < 0.0001.

To investigate the clinicopahtological features associated with a putative miR-4476/APC/β-catenin/c-Jun positive feedback loop in glioma, we performed immunohistochemistry and in situ hybridization in 87 paraffin-embedded glioma tissue samples (Supplementary Fig. 2). The clinical characteristics of the glioma patients are summarized in Table 1. We did not find a significant association between the miR-4476 expression level and patient age or gender in these 87 glioma cases. However, we observed that increased miR-4476 expression was positively correlated with tumor grade. We also confirmed that the miR-4476 expression level was negatively correlated with that of APC (Fig. 6a), but positively correlated with that of c-Jun (Fig. 6b); moreover, we also observed a negative correlation between APC and c-Jun expression (Fig. 6c).

**Table 1:** Correlation between the clinicopathologic characteristics and miR-4476 expression in glioma.

Characteristics	*n*	miR-4476 expression		*p* value
		High	Low	
Age (years)				
<45	62	28 (45.16%)	34 (54.84%)	0.2425
≥45	25	15 (60.00%)	10 (40.00%)	
Gender				
Male	51	28 (54.90%)	23 (45.10%)	0.2783
Female	36	15 (41.67%)	21 (58.33%)	
WHO grade				
I–II	41	10 (24.39%)	31 (75.61%)	<0.0001
III–IV	46	33 (71.74%)	13 (28.26%)

We then performed a Kaplan–Meier survival analysis with this glioma cohort and confirmed that patients with high miR-4476 expression had poorer overall survival (OS) rates compared with those exhibiting low miR-4476 expression (Fig. 6d). In our clinical tissue samples, patients with high APC expression tended to have longer survival times than those with low APC expression (Supplementary Fig. 3A), while high c-Jun expression appeared to be an unfavorable prognosis factor in glioma patients (Supplementary Fig. 3B). However, neither *p*-value reached significance. We suspected that nonsignificance was due to small sample size, so we reanalyzed the correlation between OS and APC expression, as well as OS and c-Jun expression, in glioma patients through the GEPIA website and CGGA database. We found that the OS rates of glioma patients were positively correlated with APC levels (Fig. 6e and Supplementary Fig. 3C) but negatively correlated with c-Jun expression (Fig. 6f and Supplementary Fig. 3D).

## Discussion

The roles of miR-4476 in glioma and the associated molecular mechanisms have not been reported to date. In this study, we found that expression of miR-4476 was increased in glioma tissues compared with that in nontumor brain tissues. We further determined that miR-4476 promotes glioma cell proliferation, migration, and invasion, and that miR-4476 levels were inversely correlated with survival in glioma patients. Combined, our data suggest that miR-4476 functions as a potential oncogenic miRNA in gliomas.

By binding to the 3’UTRs, miRNAs can repress the translation or directly induce the degradation of their target mRNAs^20,31^. Depending on the biological function of their target mRNAs, miRNAs can act as either oncogenes (onco-miRNAs) or tumor suppressors^32^. Emerging evidence has indicated that the expression of many miRNAs is dysregulated in human tumors^29,33^. Although numerous miRNAs have been shown to elicit multiple phenotypes in various types of cancers, the roles of miR-4476 in glioma progression and the associated molecular mechanisms have not been reported to date. In the present study, we found that miR-4476 is overexpressed in glioma tissues. Subsequently, we confirmed that miR-4476 promotes the proliferation, migration, and invasion of glioma cells in vitro, and enhances tumorigenicity in vivo. These results suggest miR-4476 may function as an onco-miRNA in glioma.

Aberrant Wnt/β-catenin signaling is associated with a wide range of pathologies in humans, including cancers^10,34^. As a key modulator of the Wnt/β-catenin signaling pathway, APC serves as an important tumor suppressor, especially in colorectal cancer^12,35^. A complex consisting of APC, AXIN1, and GSK3B reduces the stability of β-catenin, leading to its degradation. Reduced APC expression results in the accumulation of β-catenin and its translocation into the nucleus, where it binds to the TCF/LEF transcription factor and initiates the transcription of downstream target genes^36^. Numerous miRNAs have been reported to regulate cancer progression by altering the activation status of Wnt/β-catenin signaling^37–39^. In this study, we reveal that miR-4476 suppresses APC expression by directly targeting its 3′-UTR, thereby activating Wnt/β-catenin signaling pathway, and consequently, upregulating the expression of β-catenin, c-Jun, c-Myc, snail, slug, and CCND1. Furthermore, silencing APC reversed the suppressive effects of miR-4476 inhibitors in glioma. Together, these results support that miR-4476 promotes glioma progression by directly targeting APC and activating Wnt/β-catenin signaling.

C-Jun is an effector of many important signaling pathways like MAPK signaling and Wnt/β-catenin signaling, and has vital roles in the regulation of cell growth, proliferation, differentiation, and apoptosis^40^. As a well-studied transcription factor, c-Jun can regulate the transcription of several miRNAs through binding to their promoter regions, thereby promoting cancer progression^41,42^. Our study has demonstrated that miR-4476 activates Wnt/β-catenin signaling and upregulates c-Jun expression, which, in turn, enhances the expression of miR-4476. Further mechanistic studies showed that c-Jun could bind to the promoter region of miR-4476 and promote its transcription. Taken together, our results demonstrated the existence of a positive feedback loop involving miR-4476/APC/β-catenin/c-Jun that promotes glioma cell proliferation, migration and invasion.

Consistent with the in vitro and in vivo roles, we found that miR-4476 level was significantly increased in glioma tissue when compared to nontumor brain tissues. Moreover, patients expressing high levels of miR-4476 had shorter overall survival times than those expressing low miR-4476 levels, suggesting that increased miR-4476 expression is an unfavorable prognostic factor for glioma patients. Furthermore, miR-4476 expression was positively correlated with c-Jun expression, but negatively correlated with that of APC, which further supports the existence of a miR-4476/APC/β-catenin/c-Jun positive feedback loop in gliomas.

**Fig. 7:**
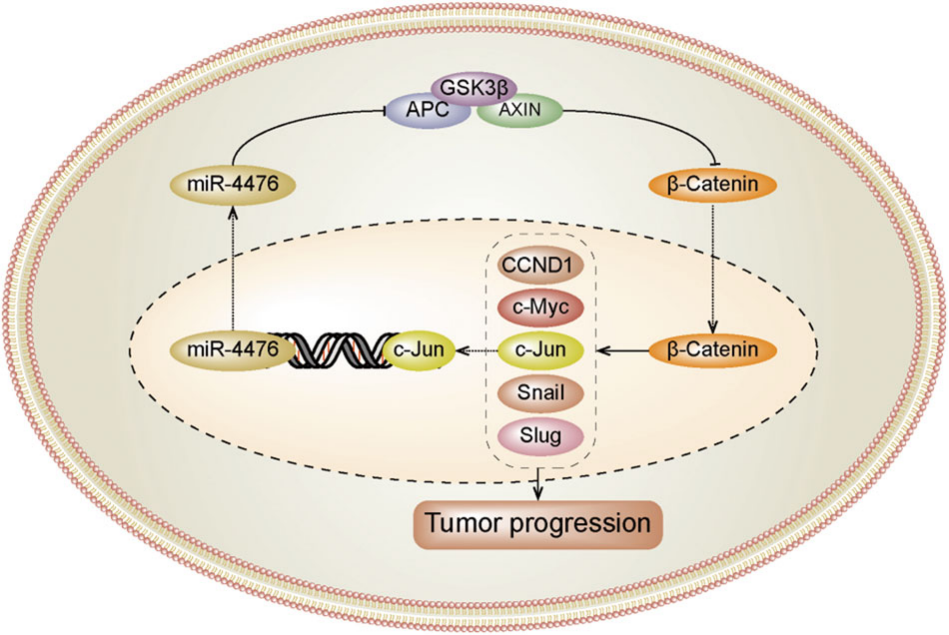
Schematic for the hypothesized atypical miR-4476/APC/β-catenin/c-Jun positive feedback loop. miR-4476 activates Wnt/β-catenin signaling by directly targeting its negative regulator APC. The activation of Wnt/β-catenin signaling in turn results in the upregulation of miR-4476 by c-Jun binding to its promotor. This feedback loop promotes glioma progression.

In conclusion, as illustrated in our working model in Fig. 7, we found that miR-4476 activates Wnt/β-catenin signaling by directly targeting its negative regulator, APC. In turn, activation of Wnt/β-catenin signaling leads to the upregulation of miR-4476 through the binding of c-Jun to the miR-4476 promoter region. Our study unveiled a novel, positive feedback mechanism through which miR-4476 promotes glioma progression, thereby providing a potential therapeutic target for the treatment of gliomas.

## Supplementary information

Supplementary Figure 1

Supplementary Figure 2

Supplementary Figure 3

Supplementary Table 1

Supplementary Table 2

Supplementary Table 3

Supplementary Table 4

clean version of supplementary figure legends
