# Supplementary figures and images for: MicroRNA-4476 promotes glioma progression through a miR-4476/APC/β-catenin/c-Jun positive feedback loop
Source: Cell Death Dis. 2020 Apr 23;11(4):269. doi: 10.1038/s41419-020-2474-4 (PMC7181615; doi:10.1038/s41419-020-2474-4)

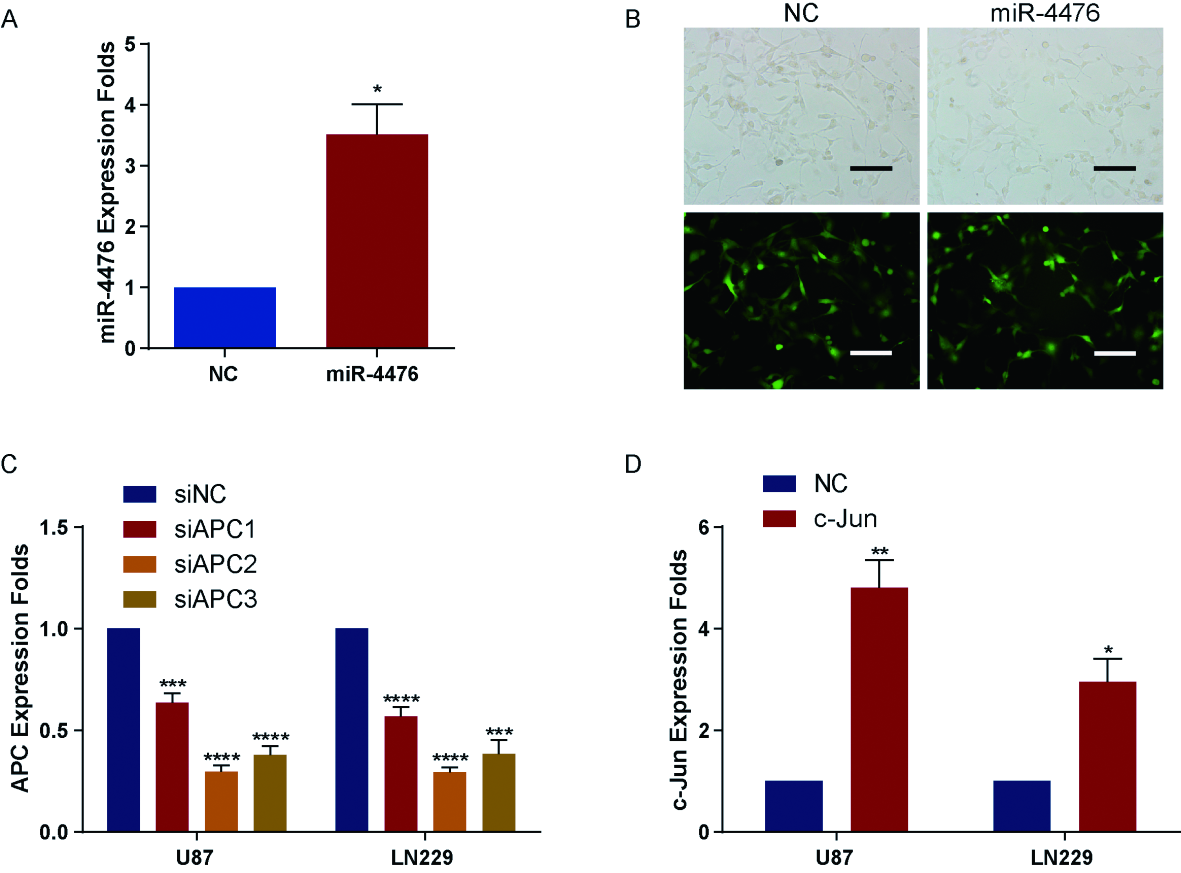

Supplement: Supplementary file 1 — Supplementary Figure 1 [file 41419_2020_2474_MOESM1_ESM.tif]

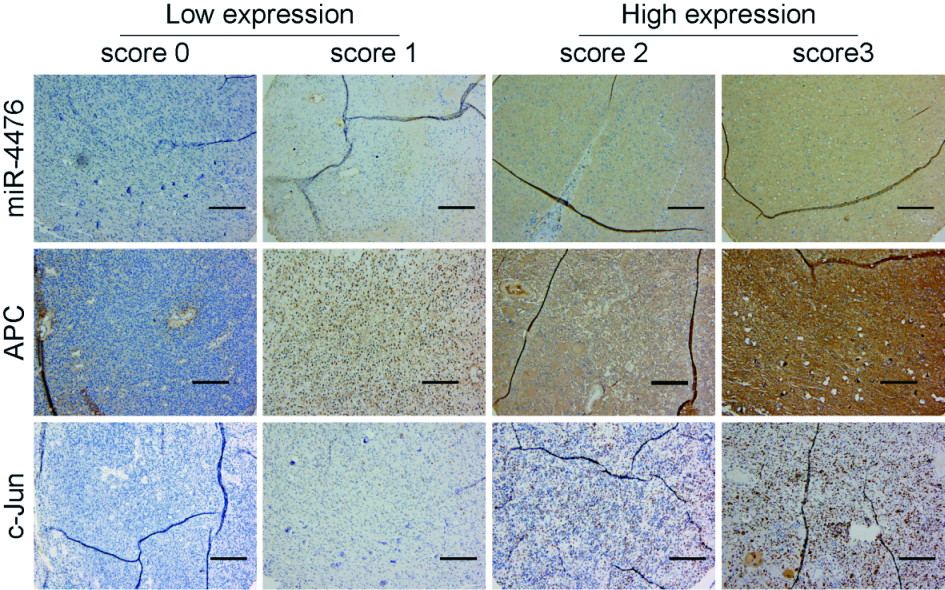

Supplement: Supplementary file 2 — Supplementary Figure 2 [file 41419_2020_2474_MOESM2_ESM.tif]

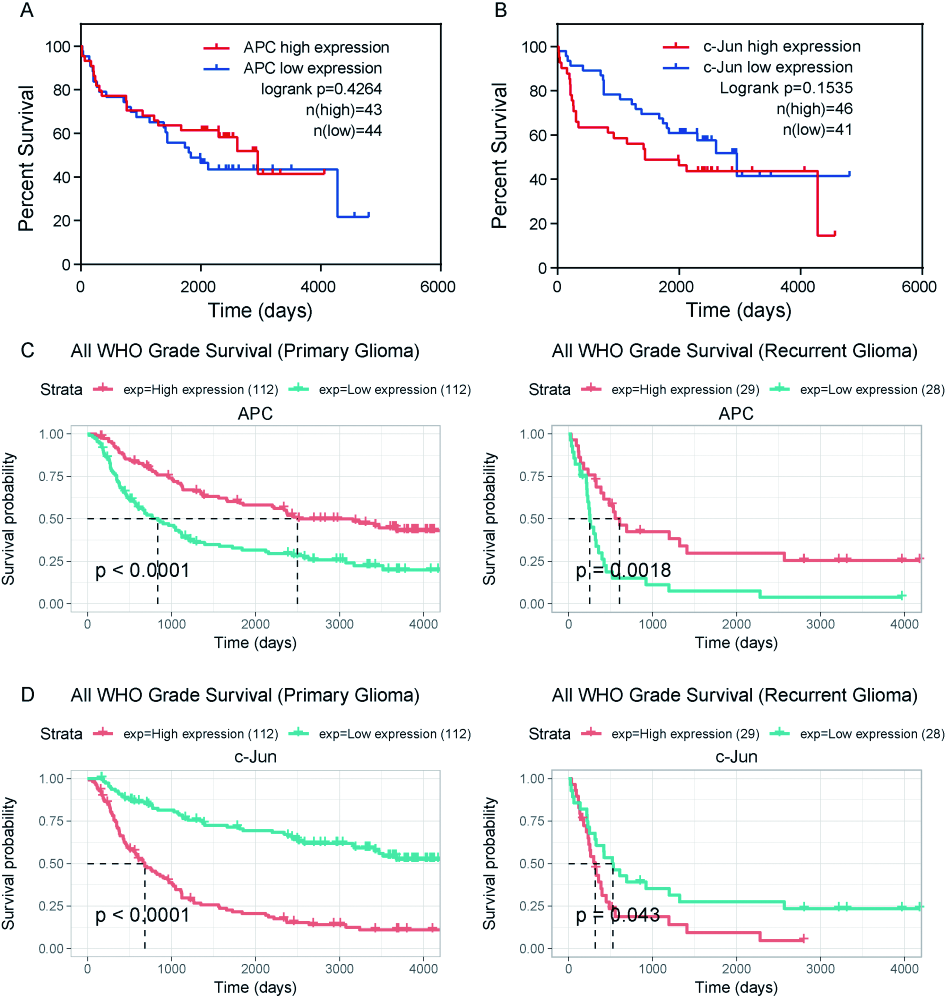

Supplement: Supplementary file 3 — Supplementary Figure 3 [file 41419_2020_2474_MOESM3_ESM.tif]
