# Supplementary material for: MicroRNA-4476 promotes glioma progression through a miR-4476/APC/β-catenin/c-Jun positive feedback loop
Source: Cell Death Dis. 2020 Apr 23;11(4):269. doi: 10.1038/s41419-020-2474-4 (PMC7181615; doi:10.1038/s41419-020-2474-4)
Supplement: Supplementary file 8 — clean version of supplementary figure legends [file 41419_2020_2474_MOESM8_ESM.docx]

**Figure Supplementary Legends：**

**Supplementary Figure 1**

(A) QPCR showing transcriptional levels of the miR-4476 with U6 used as an internal

control. (B) U87 were transfected by lentiviruses containing LV-NC or LV-miR-4476; scale bar = 50μm. (C) QPCR showing transcriptional levels of APC with ARF used as an internal control. (D) QPCR showing transcriptional levels of c-Jun with ARF used as an internal control. Mean ± S.D., **P* < 0.05, ***P* < 0.01, ****P*<0.001 and **** *P*<0.0001.

**Supplementary Figure 2**

ISH analysis of miR-4476 and IHC analysis of APC as well as c-Jun in glioma tissues; scale bar = 75μm.

**Supplementary Figure 3**

(A-B) Kaplan–Meier survival analysis of overall survival of 87 glioma patients on the basis of APC (A) or c-Jun (B) expression levels. (C-D) Kaplan–Meier survival analysis of overall survival of 224 primary glioma patients and 57 recurrent glioma patients from CGGA database on the basis of APC (C) or c-Jun (D) expression levels. Mean ± S.D., **P* < 0.05, ***P* < 0.01, ****P*<0.001 and **** *P*<0.0001.
